# How big is the effect of spinal manipulation on the pressure pain threshold and for how long does it last? – secondary analysis of data from a systematic review

**DOI:** 10.1186/s12998-019-0240-4

**Published:** 2019-04-24

**Authors:** Margaux Honoré, Charlotte Leboeuf-Yde, Olivier Gagey, Niels Wedderkopp

**Affiliations:** 1 CIAMS, University of Paris-Sud, University of Paris-Saclay, F-91405 Orsay Cedex, France; 2 CIAMS, University of Orléans, F-45067 Orléans, France; 3 Institut Franco Européen de Chiropraxie, 24 boulevard Paul Vaillant-Couturier, F-94200 Ivry sur Seine, France; 4 University of Southern Denmark, Institute for Regional Health Research, Odense, Denmark; 5 Orthopedic Department, Hospital of Southwestern Jutland, Esbjerg, Denmark

**Keywords:** Effect size, Duration, Spinal manipulation, Pressure pain threshold, Asymptomatic subjects

## Abstract

**Background:**

Spinal manipulation (SM) has been shown in a systematic review to have a statistically significant effect on the pressure pain threshold (PPT) in asymptomatic subjects, when SM is compared to a sham intervention. The magnitude and duration of this effect is unclear.

**Objectives:**

To determine the effect-size of SM in asymptomatic subjects and its duration.

**Method:**

This is a secondary analysis of data from a previous review. We sought to compare the effect-sizes in the various articles but had to calculate them ourselves, at different follow-up time measurements. Effect-sizes (Cohen’s d or Hedge’s g coefficient) were considered low, medium, and large, at the cut points of 0.2, 0.5, and 0.8, respectively.

**Results:**

Effect-sizes were reported in 6/8 studies, but all had calculated ‘within-group’ changes, not ‘between-group’ differences. Immediately after SM, only one study of four (with four measurements) had a statistically significant ‘medium’ effect size (d = 0.56; 95% CI: 00.4–1.08 to d = 0.70; 95% CI:0.18–1.22). Five minutes after SM, 4/5 studies found a statistically significant ‘medium to large’ effect-size (d = 0.51; 95% CI: 0.04–0.98 to d = 1.24; 95% CI: 0.28–2.20). Ten minutes after SM, two studies reported a ‘medium’ effect-size with statistical significance (d = 0.58; 95% CI: 0.11–1.05 to d = 0.80; 95% CI: 0.12–1.48). We drew no conclusions for the effect-sizes at one minute and thirty minutes after SM, as no between-group statistical difference was found.

**Conclusion:**

Authors need to revise their approach to ‘effect size’. Our calculations showed that the effect-size of SM on PPT may go from ‘medium’ to ‘large’ within the first five minutes but appears to diminish again within ten minutes. Research of this type should collect information for longer periods and compare results to other interventions to put results into perspective.

**Electronic supplementary material:**

The online version of this article (10.1186/s12998-019-0240-4) contains supplementary material, which is available to authorized users.

## Resume

**Contexte:**

Une revue systématique a montré que la manipulation vertébrale (MV) avait un effet statistiquement significatif sur le seuil de douleur à la pression (SDP) chez les sujets asymptomatiques, lorsque la MV était comparée à une manœuvre placébo. Cependant, l’ampleur et la durée de cet effet n’ont pas été clairement rapportées.

**Objectifs:**

Déterminer la taille d’effet de la MV chez les sujets asymptomatiques et sa durée.

**Méthode:**

Il s’agit d’une deuxième analyse des données de notre précédente revue systématique de la littérature. Nous avons cherché à comparer les tailles d’effet dans les différents articles, mais nous avons dû nous résoudre à les calculer, à différents temps de mesures. La taille d’effet rapportée (comme les coefficients *d de Cohen* ou *g de Hedge*) était considérée comme faible, moyenne et grande, aux seuils respectifs de 0,2, 0,5 et 0,8.

**Résultats:**

Nous avons constaté que 6 études sur 8 montraient le calcul de la taille d’effet, mais que toutes étaient fondées sur des changements « intra-groupe », et non sur des différences « intergroupes ». Immédiatement après la MV, seule une étude sur quatre (avec quatre mesures) présentait une taille d’effet « moyenne » statistiquement significative (d = 0,56 [IC à 95%: 00,4 à 1,08] à d = 0,70 [IC à 95%: 0,18 à 1,22]). Cinq minutes après la MV, quatre études sur cinq ont révélé une taille d’effet « moyenne à grande » statistiquement significative (d = 0,51 [IC à 95%: 0,04–0,98] à d = 1,24 [IC à 95%: 0,28-2,20]). Dix minutes après la MV, deux études ont indiqué une taille d’effet « moyenne » statistiquement significative (d = 0,58 [IC à 95%: 0,11-1,05] à d = 0,80 [IC à 95%: 0,12-1,48]). Aucune conclusion n’a été tirée des résultats une minute et trente minutes après la MV car la différence entre les groupes n’était pas statistiquement significative.

**Conclusion:**

Les auteurs doivent revoir leur approche de la « taille d’effet ». Nos calculs ont montré que l’effet de la manipulation vertébrale sur le seuil de la douleur à la pression peut passer de « moyen » à « important » dans les cinq premières minutes mais semble diminuer à nouveau dans les dix minutes. Les recherches de ce type devraient collecter des informations sur des périodes plus longues et comparer les résultats à ceux d’autres interventions afin de les mettre en perspective.

**Mots-clés:**

Taille d’effet; durée; manipulation vertébrale; seuil de douleur à la pression; sujets asymptomatiques.

## Introduction

### The effect of treatment

In order to study the validity of a treatment, it is necessary to compare its outcome with the outcome of another treatment. However, in order to test the specific *effect* of this treatment, it would be necessary to compare it with a well masked placebo.

### Measuring the effect

The effect of a treatment is, in fact, the difference between the differences in outcome in these two interventions, i.e. (i) the follow-up measurement of the treatment group minus the baseline measurement is estimated and also (ii) the follow-up measurement of the placebo group minus the baseline measurement. Thereafter, (iii) the difference between these two differences is subjected to a test for statistical significance. However, if the baseline measurements in the two groups is more or less identical, then there is no need to take the baseline measurements into account, and it would be sufficient to test only if the difference between the outcome measurements is statistically significant.

### Statistical significance vs. clinical significance

However, the statistical significance (i.e *p-value*) is not an indication of the clinical significance. The clinical significance can be judged by comparing the estimates of the treatment and placebo groups and by calculating, for example, the numbers needed to treat, in clinical studies. In experimental studies, the clinical significance would often be assessed by calculating what is called an ‘*effect size’*.

### Effect size

To show how big an effect of a particular treatment might be, one could proceed to calculate the ‘difference of differences’, as explained above. However, it would be difficult to compare studies that used different methods and units. Therefore, an index is often used, such as the Cohen’s *d* or Hedge’s *g* coefficients.

To relate the effect size to the concept of clinical significance, Cohen created a scale that can be used for either the *d* or the *g* coefficients. According to this scale, an effect size of 0.2 represents an overlap between the compared populations in their distributions of means of about 85%, which Aron [1] suggests could be compared to a difference of height between 15 and 16 years old girls, thus considered ‘small’. An effect size of 0.5 represents an overlap of about 67%, which, in the same way, could be interpreted as a difference of height between 14 and 18 years old girls, thus considered ‘medium’. An effect size of 0.8 (or above) is an overlap of 53% (or less) and represents a difference of height between 13 and 18 years old girls, thus considered ‘large’. As another example, Cohen suggests that the difference in IQ between holders of a PhD degree and a ‘typical college freshman’ is comparable to an effect size of 0.8 [2].

### Calculation of the effect size

The Cohen’s *d* coefficient is commonly calculated by the subtraction of the mean of the experimental group from the mean of the control group, and the division of this result by the standard deviation. However, this equation should be used under specific conditions, which are described extensively in the literature albeit without a clear consensus. Therefore, the effect size can be calculated in different ways and unless it is clearly described, there is room for errors and confusion. (Please, see Additional file 1 for more details).

### The regional effect of spinal manipulation on experimentally induced pain in asymptomatic subjects

#### Spinal manipulation

Spinal manipulation (SM) is used for its beneficial effect on musculoskeletal pain. It is reported to very quickly soothe musculoskeletal pain in some patients [3] but its mechanisms are not yet well understood. Nevertheless, according to a previous systematic review, SM has been reported in some studies to have a hypoalgesic effect in asymptomatic subjects exposed to experimentally induced pain, such as increasing the pressure pain threshold (PPT) [4]. However, not all studies used proper sham-controlled studies to control for the placebo effect.

#### Pressure pain threshold

Pressure pain threshold (PPT) is a type of quantitative sensory testing that can be used to understand the somato-sensory profiles of people in pain [5], but also in asymptomatic subjects [6]. It is defined as the minimal pressure which provokes a pain or a discomfort [7]. A reported increase of PPT values on subjects after a treatment would suggest an hypoalgesic action of the SM, whereas a reported decrease of PPT values would suggest hyperalgesia.

#### Spinal manipulation and its regional effect on the pressure pain threshold

To be able to establish whether SM truly has a hypoalgesic effect, we performed a systematic review in which we separated out the sham-controlled studies, as reported elsewhere [8]. A description of these studies is found in Additional file 2**.**

Thus, we found eight randomized controlled trials of good and medium quality. They investigated the regional effect of SM, compared to a sham procedure in asymptomatic subjects. As previously reported (Additional file 2), five out of these eight studies found that SM had a statistically significant effect on the PPT in these asymptomatic subjects.

However, the effect size and the duration of this effect need also to be investigated, to conclude whether this reaction is also clinically relevant.

#### The research objectives

To the best of our knowledge, the i) effect size and (ii) this effect size over time, for the PPT in asymptomatic subjects after a spinal manipulation, compared to a sham procedure, have never been reported in a systematic review. Therefore, we returned to the articles in our previous review [8] to report on these values.

## Method

### Design

This work consists of a secondary analysis of data from our previous systematic literature review, using data from eight randomized controlled trials that reported the regional effect of spinal manipulation on PPT in asymptomatic subjects compared to a sham procedure [8].

### Search strategy and extraction of data

The search strategy and extraction of data for the original review have been extensively reported (Additional file 3). The flow chart for the screening process presented in the previous review has been included in this report for information. (Please, see Additional file 4).

For the present review, a descriptive and a quality checklist were created **(**Tables 1-2**)** to fit our new objectives. The quality score for the research method in general as reported in the previous article was included in the descriptive checklist for information (Table 1).

**Table 1:** Descriptive checklist of the reported ‘effect sizes’ in eight randomized sham-controlled studies included in a previous systematic review on pressure pain threshold changes after spinal manipulation

First author Year	Quality score as reported in previous review (Honoré and al, 2018)	- Area of spinal manipulation - Regional testing site/s	Number of follow-ups and time of follow-ups	-Is there a reported effect size? (Yes/No) - Which type of “effect size” was reported (between or within-group comparison)?
Ruiz Saez 2007	8/9	- Cervical - Upper trapezius	3 follow-ups: 1) Immediately after 2) 5 min after 3) 10 min after	-Yes -Within-group comparison
Srbely2013	7.5/9	-Cervical (bilateral) - Infraspinatus muscle	4 follow-ups: 1) 1 min after 2) 5 min after 3)10 min after 4) 15 min after	-No - NA
Fernandez de la Penas 2008	7/9	- Cervical - C5-C6 level	1 follow-up: 1) 5 min after	-Yes - Within-group comparison
Fernandez de la Penas 2007	7/9	- Cervical - Ipsilateral and contralateral epicondyle	1 follow up: 1) 5 min after	- Yes - Within-group comparison
Hamilton 2007	7/9	- Cervical - Between C0 and C2	2 follow-ups: 1) 5 min after 2) 30 min after	- Yes - Within-group comparison
Yu 2012	8/9	- Lumbar - L5-S1 over apophyseal joints - L5 dermatome	1 follow-up: 1) Immediately after	- No - NA
Thomson 2009	6/9	- Lumbar -Spinous process of L3	1 follow-up: 1) Immediately after	- Yes -Within-group comparison
Fryer 2004	5/9	-Thoracic -Thoracic spinous process between T1 and T4	1 follow-up: 1) Immediately after	- Yes - Within-group comparison

NA: not applicable;

**Table 2:** Quality checklist of the reported ‘effect sizes’ in eight randomized sham-controlled studies included in a previous review on pressure pain threshold changes after spinal manipulation

First author Year	Did the authors report the between-group effect size? (Yes/No)	1) Did the authors report the ‘exact’ formula they used? (Yes/No/NA) 2) Or, at least: - Did they provide a reference? (Yes/No/NA) If so, - Was this an “exact” reference?	Did they report: 1) Number of study participants in each group 2) Exact mean values 3) Exact standard deviations	Did they report the 95% CI of their effect size, or of their within group ‘effect’ size? (Yes/No)	Was it possible to calculate between-group effect size based on data provided in the articles?
Ruiz Saez 2007	No, only the within-group comparison	1) No 2) -Yes -Yes	1) Yes 2) Yes 3) Yes	No	Yes
Srbely 2013	No, not even the within-group comparison	1) NA	1) Yes 2) Yes 3) Yes	NA	Yes
Fernandez de la Penas 2008	No, only the within-group comparison	1) No 2) -Yes - No	1) Yes 2) Yes 3) Yes	No	Yes
Fernandez de la Penas 2007	No, only the within-group comparison	1) No 2) -Yes -No	1) Yes 2) Yes 3) Yes	No	Yes
Hamilton 2007	No, only the within-group comparison	1) No 2) - Yes - No	1) Yes 2) Yes 3) Yes	No	Yes
Yu 2012	No, not even the within-group comparison	1) NA	1) Yes 2) Yes 3) Yes	NA	Yes
Thomson 2009	No, only the within-group comparison	1) No 2) - No - No	1) Yes 2) Shown in figures, so lack of precision. −3) Yes	No	Yes
Fryer 2004	No, only the within-group comparison	1) No 2) - No - No	1) Yes 2) Yes 3) Yes	No	Yes

NA: Not applicable

The present quality checklist **(**Table 2**)** is based on the various recommendations from the creator of the original effect size index [9] and its coeval authors [10], supported by more recent texts on the same subject ([2]; [11]). This consists of information on:

- whether the between-group effect size was provided [11],

- whether the formula for calculation was provided [10] or if, at least, an exact reference was provided (document and page, not just the name of a textbook), the reporting of the number of study participants, the exact mean values and standard deviations necessary to calculate the effect size [12], and the reporting of 95% confidence intervals (95% CI) [9]. If all this information was available, it would be possible to calculate the effect size.

**Fig. 1:**
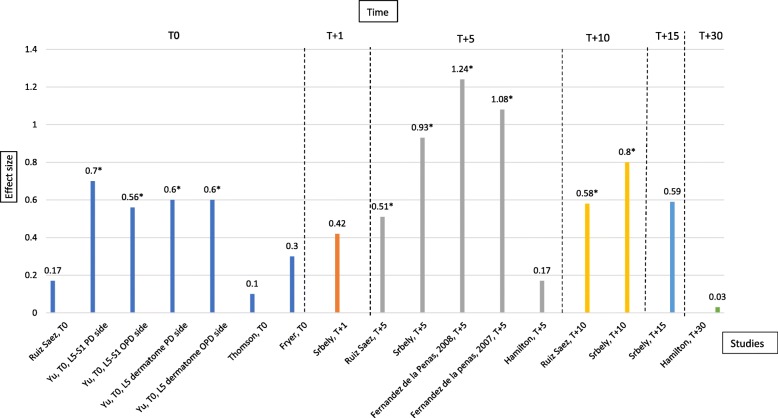
The effect size of spinal manipulation on the pressure pain threshold in asymptomatic subjects immediately after (T0), one minute after (T + 1), five minutes after (T + 5), ten minutes after (T + 10), fifteen minutes after (T + 15), and thirty minutes after (T + 30). Legend: *means statistically significant difference between-groups

Where effect size had not been calculated, it was our intention to do so, with the formulae provided in the Additional file 1 and verifying all calculations by a blinded third person. The information on the effect size at each time of measurement (provided by the authors or calculated by us) was collected in a table **(**Table 3) and illustrated in Fig. 1. We chose to report, when needed, the data concerning what happened on the “dominant side” [13], as we cared only for the regional effect.

**Table 3:** Calculation of between-group effect sizes, based on information provided in eight randomized sham-controlled studies included in a previous review on pressure pain threshold changes after spinal manipulation

First author Year	1) What is the number of participants of the experimental group (N_E_) and the control group (N_C_) and are they equal? - N_E_ = N_C_ _−_ N_E_ ≠ N_C_ 2) Are the standard deviations of the experimental group (SD_E_) and the control group (SD_C_) equal? - *SD*_*E*_ *= SD*_*C*_ *- SD*_*E*_ *≠ SD*_*C*_ 3) Which type of effect size coefficient should be used? - Cohen’s d coefficient (if N_E_ = N_C_ and *SD*_*E*_ *= SD*_*C*_ *or SD*_*E*_ *≠ SD*_*C*_*)* - Hedges’ g coefficient (if N_E_ ≠ N_C_ and *SD*_*E*_ *≠ SD*_*C*_*)* 4) Give the equations that would be used: - Effect size (*d/g*) - Standard deviation of PPT values used to calculate the effect size (SD^*^ or SD _pooled_) - Standard deviation of the effect size (SD(d)) - Confidence interval of the effect size (95% CI)	What are the reported mean PPT values for the experimental group (M_E_) and for the control group (M_C_) with their standard deviation (+/− SD), at each follow-up time (units)?	At each follow-up time, what are the: - Effect size (*d/g*), - Its standard deviation (SD(d)) - Its confidence interval (95% CI) - p value between groups	Effect sizes of clinical significant findings at each follow-up: 0.2 to 0.49 (small) - 0.5 to 0.79 (medium) - 0.8 to 1.00 (large)
Ruiz Saez 2007	1) N_E_ = N_C_ = 36 2) SD_E_ ≠ SD_C_ 3) Cohen’s d coefficient 4) -$d=\frac{M_E-{M}_C}{SD^{\ast }}$ - $\mathrm{SD}\ast =\sqrt{\frac{{SD_E}^2+{SD_C}^2}{2}}$ - $\mathrm{SD}(d)=\sqrt{\frac{N_E+{N}_C}{N_E\times {N}_C}+\frac{d^2}{2\left({N}_E+{N}_C\right)}}$ - 95% CI: [*d* − 1.96 × SD (d); *d* + 1.96 × SD(d)]	-**T0:** M_E_ = + 1.35 +/− 0.5 Kg/cm2 M_C_ = + 1.27 +/− 0.4 Kg/cm2 -**T + 5:** M_E_ = + 1.38 +/− 0.5 Kg/cm2 M_C_ = + 1.15 +/− 0.4 Kg/cm2 -**T + 10:** M_E_ = + 1.39 +/− 0.5 Kg/cm2 M_C_ = + 1.1 +/− 0.5 Kg/cm2	**-T0**: -*d* = 0.17 -SD(d) = 0.24 - 95% CI: [− 0.29; + 0.63] - *p* value: NS **-T + 5**: -d = 0.51 -SD(d) = 0.24 - 95% CI: [+ 0.04; + 0.98] - *p* < 0.01 -**T + 10**: -d = 0.58 -SD(d) = 0.24 - 95% CI: [+ 0.11; + 1.05] -p < 0.01	T0: small T + 5: medium T + 10: medium
Srbely 2013	1) N_E_ = N_C_ = 18 2) SD_E_ ≠ SD_C_ 3) Cohen’s d coefficient 4) -$d=\frac{M_E-{M}_C}{SD^{\ast }}$ -$\mathrm{SD}\ast =\sqrt{\frac{{SD_E}^2+{SD_C}^2}{2}}$ -$\mathrm{SD}(d)=\sqrt{\frac{N_E+{N}_C}{N_E\times {N}_C}+\frac{d^2}{2\left({N}_E+{N}_C\right)}}$ - 95% CI: [*d* − 1.96 × SD (d); *d* + 1.96 × SD(d)]	-**T + 1**: M_E_ = + 34.4 +/− 9.6 N M_C_ = + 30.7 +/− 7.5 N -**T + 5:** M_E_ = + 37.5 +/− 11.9 N M_C_ = + 28.7 +/− 6.0 N -**T + 10:** M_E_ = + 37.9 +/− 14.4 N M_C_ = + 28.9 +/− 6.3 N -**T + 15:** M_E_ = + 34.3 +/− 11.5 N M_C_ = + 28.6 +/− 7.0 N	**-T + 1:** -d = 0.42 -SD(d) = 0.34 - 95% CI: [− 0.24; + 1.08] -p < 0.01 -**T + 5:** -d = 0.93 -SD(d) = 0.35 − 95% CI: [+ 0.24; + 1.62] -p < 0.01 -**T + 10:** -d = 0.80 -SD(d) = 0.35 - 95% CI: [+ 0.12; + 1.48] -p < 0.01 **-T + 15:** -d = 0.59 -SD(d) = 0.34 - 95% CI: [− 0.08; + 1.26] -p < 0.01	T + 1: small T + 5: large T + 10: large T + 15: medium
Fernandez de la Penas 2008	1) N_E_ = N_C_ = 10 2) SD_E_ ≠ SD_C_ 3) Cohen’s d coefficient 4) - $d=\frac{M_E-{M}_C}{SD^{\ast }}$ - $\mathrm{SD}\ast =\sqrt{\frac{{SD_E}^2+{SD_C}^2}{2}}$ - $\mathrm{SD}(d)=\sqrt{\frac{N_E+{N}_C}{N_E\times {N}_C}+\frac{d^2}{2\left({N}_E+{N}_C\right)}}$ - 95% CI: [*d* − 1.96 × SD (d); *d* + 1.96 × SD(d)]	-**T + 5**: (dominant side/dominant side) M_E_ = + 387.6 +/− 70.9 kPa/s M_C_ = + 312.3 +/− 47.7 kPa/s	-**T + 5:** -d = 1.24 -SD(d) = 0.49 - 95% CI: [+ 0.28; + 2.20] -*p* < 0.05	T + 5: large
Fernandez de la Penas 2007	1)N_E_ = N_C_ = 15 2) SD_E_ ≠ SD_C_ 3) Cohen’s d coefficient 4) - *d* = $\frac{M_E-{M}_C}{SD^{\ast }}$ - SD^*^= $\sqrt{\frac{{SD_E}^2+{SD_C}^2}{2}}$ - SD(*d*)=$\sqrt{\frac{N_E+{N}_C}{N_E\times {N}_C}+\frac{d^2}{2\left({N}_E+{N}_C\right)}}$ - 95% CI: [*d* − 1.96 × SD (d); *d* + 1.96 × SD(d)]	-**T + 5:** M_E_ = + 2.9+/− 0.6 Kg/cm2 M_C_ = + 2.3+/− 0.5 Kg/cm2	-**T + 5:** -d = 1.08 -SD(d) = 0.48 − 95% CI: [+ 0.14; + 2.02] -p < 0.01	T + 5: large
Hamilton 2007	1) N_E_ ≠ N_C_ - N_E_ = 35 - N_C_ = 25 2) SD_E_ ≠ SD_C_ 3) Hedge’ g coefficient 4) ${\mathrm{SD}}_{\mathrm{pooled}}=\sqrt{\frac{\left({\mathrm{N}}_{\mathrm{E}}\hbox{-} 1\right){\mathrm{SD}}_{{\mathrm{E}}^2}+\left({\mathrm{N}}_{\mathrm{C}}\hbox{-} 1\right){\mathrm{SD}}_{{\mathrm{C}}^2}}{{\mathrm{N}}_{\mathrm{E}}+{\mathrm{N}}_{\mathrm{C}}\hbox{-} 2}}$ - $\boldsymbol{g}=\frac{{\boldsymbol{M}}_{\boldsymbol{E}}-{\boldsymbol{M}}_{\boldsymbol{C}}}{{\boldsymbol{SD}}_{\boldsymbol{Pooled}}}$ -$\mathrm{SD}\left(\mathrm{g}\right)=\sqrt{\frac{N_E+{N}_C}{N_E\times {N}_C}+\frac{d^2}{2\left({N}_E+{N}_C\right)}}$ - 95% CI: [*d* − 1.96 × SD (d); *d* + 1.96 × SD(d)]	-**T + 5:** M_E_ = + 398.06 +/− 133.51 kPa/s M_C_ = + 368.44 +/− 208.16 kPa/s -**T + 30**: M_E_ = + 374.58 +/− 127.50 kPa/s M_C_ = + 368.68 +/− 192.62 kPa/s	-**T + 5:** -g = 0.17 -SD(g) = 0.26 − 95% CI: [− 0.34; + 0.68] -p < 0.01 -**T + 30**: -g = 0.03 -SD(g) = 0.26 − 95% CI: [− 0.48; + 0.54] -*p* value: NS	T + 5: small T + 30: small
Yu 2012	1) N_E_ = N_C_ = 30 2) SD_E_ ≠ SD_C_ 3) Cohen’s d coefficient 4) - *d* = $\frac{M_E-{M}_C}{SD^{\ast }}$ - SD^*^= $\sqrt{\frac{{SD_E}^2+{SD_C}^2}{2}}$ - SD(*d*)=$\sqrt{\frac{N_E+{N}_C}{N_E\times {N}_C}+\frac{d^2}{2\left({N}_E+{N}_C\right)}}$ - 95% CI: [*d* − 1.96 × SD (d); *d* + 1.96 × SD(d)]	**-L5-S1 PD side** T0: M_E_ = + 5.64+/− 1.13 Kg/cm2 M_C_ = + 4.85+/− 1.12 Kg/cm2 -**L5-S1 OPD side** T0: M_E_ = + 5.56+/− 1.17 Kg/cm2 M_C_ = + 4.91+/− 1.13 Kg/cm2 -**L5 dermatome PD side** T0: M_E_ = + 4.77+/− 0.96 Kg/cm2 M_C_ = + 4.14+/− 1.13 Kg/cm2 -**L5 dermatome OPD side** T0: M_E_ = + 4.63+/− 0.95 Kg/cm2 M_C_ = + 4.09+/− 0.82 Kg/cm2	**-L5-S1 PD side** T0 -d = 0.70 -SD(d) = 0.27 − 95% CI: [+ 0.18; + 1.22] -p < 0.05 -**L5-S1 OPD side** T0 -d = 0.56 -SD(d) = 0.26 − 95% CI: [+ 0.04; + 1.08] -p < 0.05 - **L5 dermatome PD side** T0 -d = 0.60 -SD(d) = 0.26 − 95% CI: [+ 0.08; + 1.12] -p < 0.05 - **L5dermatome OPD side** T0 -d = 0.60 -SD(d) = 0.26 − 95% CI: [+ 0.08; + 1.12] -p < 0.05	**-L5-S1 PD side** T0: medium -**L5-S1 OPD side** T0: medium - **L5 dermatome PD side** T0: medium - **L5dermatome OPD side** T0: medium
Thomson 2009	1) N_E_ ≠ N_C_ - N_E_ = 19 - N_C_ = 13 2) SD_E_ ≠ SD_C_ 3) Hedge’g coefficient 4) - *g* = $\frac{M_E-{M}_C}{SD_{Pooled}}$ ${\mathrm{SD}}_{\mathrm{pooled}}=\sqrt{\frac{\left({\mathrm{N}}_{\mathrm{E}}\hbox{-} 1\right){\mathrm{SD}}_{{\mathrm{E}}^2}+\left({\mathrm{N}}_{\mathrm{C}}\hbox{-} 1\right){\mathrm{SD}}_{{\mathrm{C}}^2}}{{\mathrm{N}}_{\mathrm{E}}+{\mathrm{N}}_{\mathrm{C}}\hbox{-} 2}}$ -$\mathrm{SD}\left(\mathrm{g}\right)=\sqrt{\frac{N_E+{N}_C}{N_E\times {N}_C}+\frac{d^2}{2\left({N}_E+{N}_C\right)}}$ - 95% CI: [*d* − 1.96 × SD (d); *d* + 1.96 × SD(d)]	(Approximate data) -**T0**: M_E_ = + 2.2 +/− 1.1 Kg/cm2 M_C_ = + 2.1 +/− 0.8 Kg/cm2	**-T0**: -g = 0.10 -SD(g) = 0.36 − 95% CI: [− 0.61; + 0.81] -p value: NS	T0: small
Fryer 2004	1) N_E_ = N_C_ = 32 2) SD_E_ ≠ SD_C_ 3) Cohen’s d coefficient 4) -$d=\frac{M_E-{M}_C}{SD^{\ast }}$ -$\mathrm{SD}\ast =\sqrt{\frac{{SD_E}^2+{SD_C}^2}{2}}$ -$\mathrm{SD}\left(\mathrm{g}\right)=\sqrt{\frac{N_E+{N}_C}{N_E\times {N}_C}+\frac{d^2}{2\left({N}_E+{N}_C\right)}}$ - 95% CI: [*d* − 1.96 × SD (d); *d* + 1.96 × SD(d)]	-**T0**: M_E_ = + 216.51 +/− 90.50 kPa M_C_ = + 244.64 +/− 91.59 kPa	**-T0**: -d = 0.30 -SD(d) = 0.25 − 95% CI: [− 0.19; + 0.79] -p value: NS	T0: medium

NS: not significant; T0: Values at baseline; T + 1: Values after one minute; T + 5: Values after five minutes; T + 10: Values after ten minutes; T + 15: Values after fifteen minutes; T + 30: Values after thirty minutes; PD: Pelvic Deficiency; OPD: Opposite Pelvic Deficiency

### Data analysis

Effect sizes were calculated using Eqs. 1 to 7 (as described in Additional file 1). The effect sizes and their 95% CI were calculated with Microsoft® Excel, version 16.17 (180909). Statistical significance of the effect size was defined as when the 95% CI does not include ‘zero’ [14]. The effect size was defined as small (*d < 0.5*), medium (*0.5 < d < 0.8*) or large (d > 0.8) [9].

## Results

### General description of the studies and their reported effect size

These studies have been extensively described in our previous review [8], with a general description available in Additional file 2. Briefly, the eight studies included in our new analyses provided information on the PPT at different follow-up times; four studies immediately after, one study one minute after, five studies five minutes after, two studies ten minutes after, one study fifteen and thirty minutes after the interventions. The quality of the eight studies was established in the previous review to range from ‘medium’ to ‘good’ (Table 1).

In the present review, no additional quality score for the effect size was given, as no definitive consensus can be found on this subject. However, we noted that no study reported a between-group effect size. Instead, they reported ‘effect sizes’ of intra-group differences, i.e. in fact, the ‘outcome sizes’. Further, two studies failed to report any effect size at all (Table 1). In addition, no study reported the exact formula they used, and only one provided a ‘precise’ reference (Table 2). No additional descriptive information on the ‘effect size’ was given (95% CI or SD (*d*)). In sum, the reported ‘effect sizes’ were not real effect sizes, not transparent and possibly not comparable.

Fortunately, all studies had exploitable information, with reported numbers of study participants in each group, exact mean values and exact standard deviations, and only one study provided values only in a figure, which made it possible to retrieve information although it, for this reason, lacked precision. Thus, all authors had provided sufficient information to make it possible for us to calculate their between-group effect sizes (Table 3**).**

### Our calculations of effect sizes at each follow-up time

#### Effect size immediately after spinal manipulation

Four studies had effect sizes immediately after SM, ranging from small [15–17] to medium [18] but only one study (with four different measurements) found these to be statistically significant [18], ranging from d = 0.56 (95% CI: 0.04–1.08) to d = 0.70 (95% CI: 0.18–1.22). In sum, the immediate effect would be considered as of medium size.

#### Effect size one minute after spinal manipulation

One study [19] was found to have a non-significant and small effect size one minute after SM (d = 0.42, 95% CI: - 0.24-1.08) We drew no conclusion for this time interval.

#### Effect size five minutes after spinal manipulation

Five studies provided information on effect size five minutes after SM. One study [20] had a small non-significant effect size (g = 0.17, 95% CI: -0.34-0.68) whereas one [15] was classified as medium (d = 0.51, 95% CI: 0.04–0.98) and three as large [13, 19, 21] (from d = 0.93, 95% CI: 0.24–1.08 to d = 1.24, 95% CI: 0.28–2.20) and all statistically significant. In general, the effect at this time can therefore be considered mainly large.

#### Effect size ten minutes after spinal manipulation

Two studies had data that could be transformed into ten minutes effect sizes, [15] defined as medium (d = 0.58, 95% CI: 0.11–1.05) and the other [19] as borderline large (d = 0.80, 95% CI: 0.12–1.48), both statistically significant. This effect at ten minutes is therefore considered medium.

#### Effect size fifteen minutes after spinal manipulation

One study [19] found a non-significant, medium effect size fifteen minutes after SM (d = 0.59, 95% CI: - 0.08-1.26). No conclusion was drawn on this result.

#### Effect size thirty minutes after spinal manipulation

One study [20] had a non-significant, small effect size thirty minutes after SM (g = 0.03; 95% CI: - 0.48-0.54)*.* No conclusion was drawn on this, but it is likely that the effect is no more present.

The results are illustrated in Fig. 1.

## Discussion

### Summary

In this additional analysis of data from a previous systematic review, we were confused by the reported effect sizes. A systematic approach revealed that no study reported between-group size differences, instead using the within-group differences, when reported at all. Further, none provided details on how this ‘effect size’ had been calculated. Therefore, we used information provided in the reviewed articles to produce our own estimates.

According to our own calculations obtained from data available in the eight reviewed studies, the estimated effect size of spinal manipulation on the PPT in asymptomatic subjects is ‘medium’ immediately after the intervention (T0), ‘mainly large’ five minutes after (T + 5) and ‘mainly medium’ ten minutes after the intervention (T + 10). No certain estimation of the effect size can be reported beyond T + 10, but it may be small after 30 min.

Using the examples provided in the introduction [1, 2] to explain the clinical importance of the different effect sizes, the ‘medium’ effect size immediately after SM would thus correspond to a difference in height between 14 and 18 years old girls. The ‘large’ effect size five minutes after SM would correspond to the difference in IQ between holders of a PhD degree and a ‘typical college freshman’. The ‘medium’ effect size ten minutes after the intervention would, again, correspond to a difference in height between 14 and 18 years old girls.

The effect of SM on the PPT in asymptomatic subjects is therefore reported to be a reasonably large but probably short-lasting phenomenon. Whether these changes can also be ‘appreciated’ by study subjects, in such a way that they can differentiate between a small, medium and large effect size, is not known. Nevertheless, it serves as a comparator with other interventions in the same domain. For example, it could be used to compare the effect over time or effects of different types of interventions.

### Methodological considerations of our own review

A description of the studies is found in the Table 1. Our quality checklist was established according to the various recommendations in the literature, including those provided by the creator of the Cohen’s d coefficient. There is no definitive consensus on the calculation of the effect size nor on how to assess its quality, so we did not judge the quality of work in the reviewed articles but used our systematic approach to obtain a general understanding of the various effect size values (Table 2). This can be done online to obtain the effect size with its SD (d) and 95% CI, with A Practical Meta-Analysis Effect Size Calculator [22]. A blinded third researcher verified the calculations. Obviously, this approach assumes that the groups that are being compared are fairly similar at base-line. We did not investigate if this was the case.

### Other methods of reporting the treatment effect

Depending on the type of data (continuous or categorial), treatment effect can be reported in other ways than with Cohen’s d. Relative Risk, Odds Ratio, Number Needed to Treat (NNT) and Area Under the Curve are other possibilities [23].

### Recommendations regarding effect size reporting

This additional analysis of data from our previous review on the effect of spinal manipulation on the pressure pain threshold in asymptomatic subjects revealed that all reviewed studies that reported an effect size used the within-group rather than the between-group differences. However, the within-group effect size is a purely descriptive outcome, interesting perhaps to understand the full picture of an effect, but it should never be provided alone. Therefore, the between-group calculations need to be calculated properly and in a transparent manner, to ensure that they are correct and comparable to other reports. We provide some information on how to do this in our Additional file 1, and we also provide references for these calculations.

## Conclusion

The effect of spinal manipulation on the pressure pain threshold in asymptomatic subjects, as calculated by us, is ‘medium’ immediately after the intervention, has increased to mainly ‘large’ five minutes after and descended to mainly ‘medium’ ten minutes after intervention. The potential effect should be investigated over a longer period of time, and for other comparable interventions, to confirm if this effect is indeed only short lasting and to put it into a clinical perspective.

## Additional files

Additional file 1: Calculation of the effect size [24–27]. (DOCX 70 kb)

Additional file 2: General description of data extracted from the abstracts of eight randomized controlled trials on the regional effect of spinal manipulation on the pressure pain threshold in asymptomatic subjects. (DOCX 20 kb)

Additional file 3: Search equation, inclusion and exclusion criterion of the previous review. (DOCX 14 kb)

Additional file 4: PRISMA flow-chart of the previous review. (DOCX 88 kb)

## Abbreviations

HVLA: High Velocity Low Amplitude
PPT: Pressure Pain Threshold
SM: Spinal manipulation
