# Supplementary material for: How big is the effect of spinal manipulation on the pressure pain threshold and for how long does it last? – secondary analysis of data from a systematic review
Source: Chiropr Man Therap. 2019 Apr 24;27:22. doi: 10.1186/s12998-019-0240-4 (PMC6480891; doi:10.1186/s12998-019-0240-4)
Supplement: Supplementary file 1 — Calculation of the effect size [24–27]. (DOCX 70 kb) [file 12998_2019_240_MOESM1_ESM.docx]

**Additional file 1**

*Calculation of the effect size index*

There are several ways to calculate the “between-group” effect size, as reported by Ellis and al in 2009 [24], but all with specific conditions of utilization. The most commonly reported is the Cohen’s *d* coefficient [25], which is the subtraction of the mean of the experimental group from the mean of the control group (M_E_-M_C_), and the division of the result by the standard deviation (SD) (*Equation 1*). However, the utilization of this equation is on the condition of a homogeneity of both the number of participants per group (N_E_=N_C_) and of their standard deviations (SD_E_=SD_c_).

*Equation 1*: Cohen’s *d* = $\frac{\left( M_{E}-M_{C} \right)}{SD}$

When this is not the case, one of two scenarios can be selected.

1. When the sizes of each population remain equal (N_E_=N_C_), but with different SDs

(SD_E_ ≠ SD_C_**)**, then a different calculation of a pooled SD (SD^*^) is required [24] (*Equation 2***)**.

*Equation 2*: SD^*^= $\sqrt{\frac{{{SD}_{E}}^{2}+{{SD}_{C}}^{2}}{2}}$

2) When, on the other hand, the size of each population is not equal (N_E_ ≠ N_C_**),** also with different SDs **(**SD_E_ ≠ SD_C_**),** then the Hedges’ g coefficient could be used, according to [24] (*Equation 3***)** with yet another calculation of a pooled SD (SD _Pooled_) (*Equation 4***)** [26]


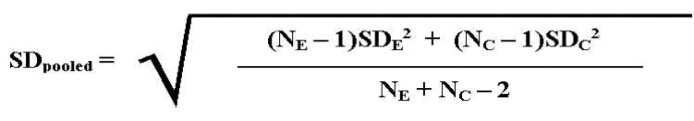
*Equation 3*: Hedge’s *g* = $\frac{\left( M_{E}-M_{C} \right)}{{SD}_{Pooled}}$

*Equation 4:*

The Hedges’*g* coefficient can also be calculated from the Cohen’s *d* coefficient [24] (*Equation 5*).

*Equation 5*: *g* = *d* (1- $\frac{3}{4\left( N_{E}+N_{c} \right)-9}$)

Finally, it is not enough only to report the effect size (Cohen’s *d* or Hedges’*g* coefficients) [9]. Indeed, its 95% interval of confidence **(**95% CI**)** **(***Equation 6*) and standard deviation (SD (d)) *(Equation 7)* should also be reported [27].

*Equation 6:* 95% CI: [*d* − 1.96 × SD (*d*); *d* + 1.96 × SD (*d*)]

*Equation 7*: SD (*d*) =$\sqrt{\frac{N_{E}+N_{C}}{N_{E} \times N_{C}}+\frac{d^{2}}{2(N_{E}+N_{C})}}$

References :

9. Cohen, J. (1994). **The earth is round (p < .05).** American Psychologist, 49(12), 997-1003

24. Ellis, P.D. (2009). “**Effect size equations**,” website: [http://www.polyu.edu.hk/mm/effectsizefaqs/effect_size_equations2.html] accessed on [*September 2018*]

25. Cohen. **Statistical power analysis for the behavioral sciences**. 2nd edition. 1988. Page 43

26. *Hedge LV, Olkin .* **Statistical methods for meta-analysis***. Orlando, Academic Press Inc. 2014, p.86*

27. Hedges LV. **Distribution theory for Glass’s estimator of effect size and related estimators**. *Journal of Educational Statistics*, 1981. 6(2):106-128. Page 110. 🡪 27
