# Supplementary material for: How big is the effect of spinal manipulation on the pressure pain threshold and for how long does it last? – secondary analysis of data from a systematic review
Source: Chiropr Man Therap. 2019 Apr 24;27:22. doi: 10.1186/s12998-019-0240-4 (PMC6480891; doi:10.1186/s12998-019-0240-4)
Supplement: Supplementary file 2 — General description of data extracted from the abstracts of eight randomized controlled trials on the regional effect of spinal manipulation on the pressure pain threshold in asymptomatic subjects. (DOCX 20 kb) [file 12998_2019_240_MOESM2_ESM.docx]

**Additional file 2**

General description of eight randomized controlled trials on the regional effect of spinal manipulation on the pressure pain threshold in asymptomatic subjects, with the texts extracted from their abstracts

| **First author**  **Year** | **Objectives** | **Subjects** | **Interventions** | **Follow-up(s) after baseline** | **Conclusion** |
| --- | --- | --- | --- | --- | --- |
| Ruiz Saez  2007 | This study analyzed the immediate effects on pressure pain threshold (PPT) in latent myofascial trigger points (MTrPs) in the upper trapezius muscle of a single cervical spine manipulation directed at the C3 through C4 level. | Seventy-two volunteers (27 men and 46 women; mean age, 31 years; SD, 10 years) participated in this study. | Subjects were divided randomly into 2 groups: manipulative group, which received a cervical spine manipulation directed at the C3 through C4 level, and a placebo group, which received a sham manual procedure. | 1, 5, and 10 minutes | The results suggest that a cervical spine manipulation directed at the C3 through C4 segment induced changes in pressure pain sensitivity in latent MTrPs in the upper trapezius muscle. |
| Srbely  2013 | The purpose of this study was to investigate if spinal manipulative therapy (SMT) can evoke immediate regional antinociceptive effects in myofascial tissues by increasing pressure pain thresholds (PPTs) over myofascial trigger points in healthy young adults. | A total of 36 participants (19 men, 17 women; age, 28.0 [5.3] years; body mass index, 26.5 [5.7] kg/m(2)) with clinically identifiable myofascial trigger points in the infraspinatus and gluteus medius muscles were recruited from the University of Guelph, Ontario, Canada | Participants in the test group received chiropractic SMT targeted to the C5-C6 spinal segment. Participants in the control group received sham SMT. | 1, 5, 10, and 15 minutes | This study showed that SMT evokes short-term regional increases in PPT within myofascial tissues in healthy young adults. |
| Fernandez de la Penas  2008 | This study examines if C7-T1 manipulation results in changes in pressure pain thresholds (PPT) over bilateral C5-C6 zygapophyseal joints in asymptomatic subjects. | Thirty subjects, 13 men and 17 women, without a current history of neck, shoulder, or upper extremity pain participated | Participants were randomly divided into 3 groups: experimental dominant group, subjects who received the manipulative thrust directed at the right side of the C7-T1 joint; experimental nondominant group, those who received the thrust on the left side of the C7-T1 joint; and a placebo group, those who received a sham-manual procedure. | 5 minutes | These results suggest that a C7-T1 manipulation induced changes in PPT in both right and left C5-C6 zygapophyseal joints in healthy subjects |
| Fernandez de la Penas  2007 | To compare the immediate effects on pressure pain threshold (PPT) tested over the lateral elbow region following a single cervical high-velocity low-amplitude (HVLA) thrust manipulation, a sham-manual application (placebo), or a control condition; and to analyze if a different effect was evident on the side ipsilateral to, compared to the side contralateral to, the intervention. | Fifteen asymptomatic volunteers (7 male, 8 female; aged 19-25 years) participated in this study. | Each subject attended 3 experimental sessions on 3 separate days, at least 48 hours apart. At each session, subjects received either the manipulation, placebo, or control intervention provided by an experienced therapist. The manipulative intervention was directed at the posterior joint of the C5-6 vertebral level. | 5 minutes | The application of a manipulative intervention directed at the posterior joint of the C5-6 vertebral level produced an immediate increase in PPT over the lateral epicondyle of both elbows in healthy subjects. Effect sizes for the HVLA thrust manipulation were large, suggesting a strong effect of unknown clinical importance at this stage, whereas effect sizes for both placebo and control procedures were small, suggesting no significant effect. |
| Hamilton  2007 | The aim of this controlled, single blinded study was to investigate whether HVLA manipulation of the occipito–atlantal (OA) joint and/or an MET stretch had an effect on pressure pain threshold (PPT) in the suboccipital musculature in an asymptomatic population. | Participants (N = 90; mean age = 23 ± 5; 29 males and 61 females) | Participants were randomly allocated into three intervention groups and then received an HVLA thrust to cavitate the right and left OA joints, an MET stretch applied to the suboccipital muscles bilaterally, or a sham ‘functional’ technique | 5 and 30 minutes | Neither HVLA manipulation nor MET significantly changed the PPT of the suboccipital muscles in asymptomatic participants. Both techniques produced greater mean increases in PPT and effect sizes compared to the control group, and investigation of the effect of these techniques in a symptomatic population is recommended. |
| Yu  2012 | The purpose of this study was to investigate the effects of instrument-assisted spinal manipulative therapy (SMT) targeted to the low-back region on changes in pressure pain thresholds (PPTs) and basal electromyographic activity (BEA) in asymptomatic participants. | 30 participants, 19 men and 11 women (mean age, 24.5±3.9 years), without a current history of low-back pain. | Each participant attended all 2 treatment group sessions and received instrument-assisted SMT or a sham manipulation procedure. Instrument-assisted SMT was administered using the Activator Method protocol. | Immediately after SMT | The application of instrument-assisted SMT resulted in an immediate and widespread hypoalgesic effect with local muscle relaxation in asymptomatic participants. |
| Thomson  2009 | To compare changes in pressure pain threshold (PPT) following spinal high-velocity low-amplitude thrust manipulation (HVLAT) and spinal mobilisation. | Fifty asymptomatic subjects (mean age 27 (6) years; 29 males and 21 females) volunteered to participate in a randomised controlled, singled blinded design study. | Subjects were screened for suitability and were randomly allocated into one of three intervention groups where they received either a unilateral spinal HVLAT or a spinal mobilisation of the lumbar spine, or a sham ‘laser’ procedure (control). | Immediately after HVLAT | Neither spinal HVLAT nor mobilisation had a significant effect on PPT of the lumbar spine in asymptomatic subjects. Only spinal mobilisation appeared to have a greater mean increase in PPT and effect size than the control group. Further investigation into the hypoalgesic effects of these techniques on symptomatic subjects is suggested. |
| Fryer  2004 | The aim of this controlled, single blinded study was to investigate the effect of manipulation and mobilisation on pressure-pain thresholds in the thoracic spine in an asymptomatic population. | Subjects (n=96) | Subjects were randomly allocated into three intervention groups, and received either a single high velocity extension thrust, thirty seconds of extension mobilisation, or thirty seconds of sham treatment (control) consisting of simulated ‘laser acupuncture’. | Immediately after manipulation | Both manipulation and mobilisation produced significantly increased pressure-pain thresholds (decreased sensitivity to pressure) in the thoracic spine, whereas the sham treatment did not. Mobilisation appeared to be more effective than manipulation for increasing pressure-pain thresholds when applied to the thoracic spine in asymptomatic subjects. |
