# Supplementary material for: How big is the effect of spinal manipulation on the pressure pain threshold and for how long does it last? – secondary analysis of data from a systematic review
Source: Chiropr Man Therap. 2019 Apr 24;27:22. doi: 10.1186/s12998-019-0240-4 (PMC6480891; doi:10.1186/s12998-019-0240-4)
Supplement: Supplementary file 3 — Search equation, inclusion and exclusion criterion of the previous review. (DOCX 14 kb) [file 12998_2019_240_MOESM3_ESM.docx]

**Additional file 3**

*Search strategy*

We did a systematical literature search of PubMed, Embase and Cochrane using the search terms [(spinal manipulation) **AND** (experimental pain)]; [(spinal manipulative therapy **OR** spinal manipulation) **AND** ((experimental pain **OR** quantitative sensory testing **OR** pressure pain threshold **OR** pain threshold)]. We generally used the same search terms as in a previous systematic review conducted on the same topic [4]. We also searched reference lists of relevant articles including those in the previous review [4]. The present search was performed between 2011.01.01 and 2017.06.13.

The inclusion criteria were SM performed manually and/or mechanically assisted, anywhere in the spine; the use of PPT, in an asymptomatic region and on the same day as SM; reported in experimental studies with at least one external or internal control group. Studies on only spinal motion or tenderness, other reviews, case reports and studies with less than 15 invited participants in each group were excluded.

Data from this search were reported previously [8] by us and the present review uses information from that review.
