# Supplementary material for: How big is the effect of spinal manipulation on the pressure pain threshold and for how long does it last? – secondary analysis of data from a systematic review
Source: Chiropr Man Therap. 2019 Apr 24;27:22. doi: 10.1186/s12998-019-0240-4 (PMC6480891; doi:10.1186/s12998-019-0240-4)

**Additionnal file 4**

We used the preferred reporting item for systematic reviews and meta-analysis (PRISMA) flow chart to record our screening of the articles for inclusion and to report the flow of the review.


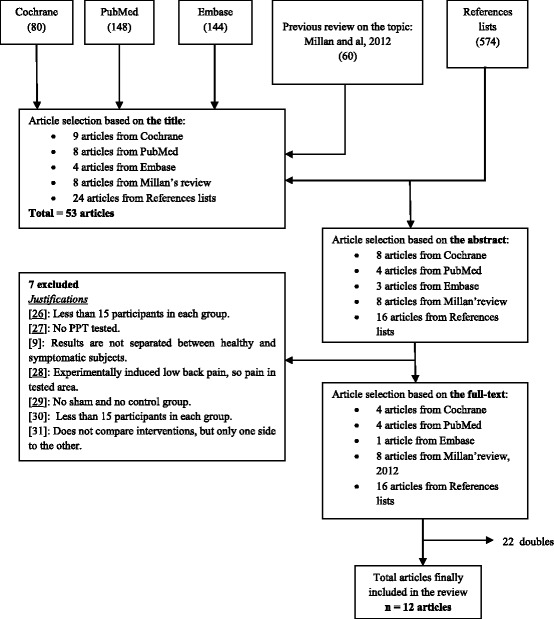

Supplement: Supplementary file 4 — PRISMA flow-chart of the previous review. (DOCX 88 kb) [file 12998_2019_240_MOESM4_ESM.docx]
